# In patients with chronic aplastic anemia, bone marrow–derived MSCs regulate the Treg/Th17 balance by influencing the Notch/RBP-J/FOXP3/RORγt pathway

**DOI:** 10.1038/srep42488

**Published:** 2017-02-14

**Authors:** Hongbo Li, Lin Wang, Yan Pang, Zujun Jiang, Zenghui Liu, Haowen Xiao, Haijia Chen, Xiaohu Ge, Hai Lan, Yang Xiao

**Affiliations:** 1 Department of Hematology, General Hospital of Guangzhou Military Command of Chinese PLA; Guangzhou, Guangdong 510010, P.R. China; 2 Department of Emergency, The First Affiliated Hospital of Guangzhou University of Chinese Medicine; Guangzhou, Guangdong 510405, P.R. China; 3 Guangdong Saliai Stem Cell Research Institute, Guangzhou, Guangdong, 510000, P.R. China; 4 Department of Hematology, The First Affiliated Hospital of Guangzhou University of Chinese Medicine, Guangzhou, Guangdong 510405 P.R. China

## Abstract

The standard treatment for aplastic anemia (AA) in young patients is a matched sibling hematopoietic stem cell transplant. Transfusion of a chronic AA patient with allogeneic bone marrow–derived mesenchymal stromal cells (BMMSCs) is currently being developed as a cell-based therapy, and the safety and efficacy of such transfusions are being continuously improved. Nevertheless, the mechanisms by which BMMSCs exert their therapeutic effects remain to be elucidated. In this study, mesenchymal stromal cells (MSCs) obtained from bone marrow donors were concentrated and intravenously injected into 15 chronic AA patients who had been refractory to prior immunosuppressive therapy. We showed that BMMSCs modulate the levels of Th1, Th2, Th17 and Treg cells, as well as their related cytokines in chronic AA patients. Furthermore, the percentages of Th1 and Th17 cells among the H-MSCs decreased significantly, while the percentage Treg cells increased. The Notch/RBP-J/FOXP3/RORγt pathway was involved in modulating the Treg/Th17 balance after MSCs were transfused *in vitro*. Additionally, the role played by transfused MSCs in regulating the Treg/Th17 balance via the Notch/RBP-J/FOXP3/RORγt pathway was further confirmed in an AA mouse model. In summary, in humans with chronic AA, BMMSCs regulate the Treg/Th17 balance by affecting the Notch/RBP-J/FOXP3/RORγt pathway.

Acquired AA is thought to be a disorder caused by an immune-mediated attack against hematopoietic stem and progenitor cells. This attack results in immune-mediated bone marrow failure characterized by signs of hypoplasia, pancytopenia, and fatty bone marrow^1^,^2^. The current collective data suggest infused MSCs as a promising tool for treating immune-based disorders. This is due to their capacity to modulate immune responses, support hematopoiesis, differentiate into several tissues types, produce cytokines, and repair tissue^3^.

Previous investigators reported that allogeneic MSCs can be safely infused into AA patients, and promote hematopoietic recovery in such patients^4^,^5^. Furthermore, the percentage of CD4^+^CD25^+^FOXP3^+^Treg cells in the peripheral blood of AA patients was significantly lower than the percentage in normal healthy subjects. While an MSC transfusion may promote hematopoietic recovery and improve hematopoiesis by modulating the inflammatory microenvironment and distribution of T-cell subtypes^4^, an imbalance of Th1 and Th2 cells is thought to be involved in the immune-mediated destruction of bone marrow in chronic AA patients^6^. The mechanisms by which BMMSCs regulate the Treg/Th17 cell balance in an AA environment remain to be elucidated.

Tregs, a specialized T cell lineage, have an indispensable function in the control of immunological unresponsiveness to self-antigens and immune responses deleterious to the host^7^.

Collective date showed that Tregs have been identified as dedicated suppressors of diverse immune responses and inflammation, and central keepers of peripheral tolerance^8^. Th17 cells have been characterized as a novel subset of CD4^+^T cells that produce interleukin-17 and serve as immune effectors in various settings, including inflammation, infection, and autoimmunity^9^. Notoriety of Th17 cells driven by IL-23, were major contributors to autoimmune inflammation. Increasing data implicates Treg and Th17 subsets have opposing roles in immunity regulation and the generation and balance of two subsets cells were regulated by a balance of transcription factors governing CD4+ T cell differentiation^10^. In this study, BMMSCs were intravenously infused into 15 chronic AA patients, and the results showed that BMMSCs modulate the levels of Th1, Th2, Th17 and Treg cells, as well as their related cytokines in chronic AA patients.

The Notch signalling pathways comprise an evolutionarily conserved cell-to-cell communication system that controls cell proliferation, specifications, and cell fate during both embryonic development and adult life^11^. An increasing amount of data suggests that the Notch pathways play differential roles in regulating the differentiation and function of Th1, Th2, Th17, and Treg cells^7^,^8^,^10^,^12^,^13^,^14^. Notchl, Notch2, Notch3, and Notch4 are Notch signaling receptors, while Dll1 Dll2, Dll3, Jaggedl, and Jagged2 are Notch signaling ligands^11^. The retinoid-related orphan receptor (RORγt) and forkhead box P3 (FOXP3) are specific transcription factors found in Th17 and Treg cells, respectively. Recombination signal binding protein for the immunoglobulin kappa J region (RBP-J) is a Notch effector protein that plays an important role in the Notch/RBP-J pathway^15^. The expression of Notchl, Notch2, Dll1, Jaggedl, RBP-J, and Foxp3 in the PBMCs of AA patients after a MSC infusion were still unclear.

Our present study shows that BMMSC transfusion decreased the percentages of Th1 and Th17 cells and increased the percentage of Treg cells in patient peripheral blood significantly.

Additionally, *In vitro*, the role of transfused MSCs in regulating the Treg/Th17 balance via the Notch/RBP-J/FOXP3/RORγt pathway was further confirmed in an AA mouse model.

## Results

### BMMSCs modulated Th1 and Th17/Treg cell differentiation in chronic AA patients

**Figure 1:**
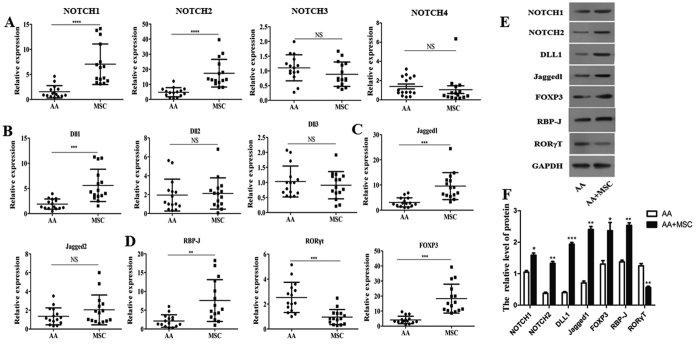
BMMSCs modulated the differentiation of Th1 and Th17/Treg cells in chronic AA patients. MSCs (mean number = 6 × 10^5^ cells/kg) were intravenously injected into 15 chronic AA patients who had been refractory to prior immunosuppressive treatment. (**A**) Levels of the related cytokines (IL-2, INF-γ, TNF-α, and TGF-β) in bone marrow were measured by ELISA at one month after treatment with MSCs. (**B**) Serum levels of the related cytokines (IL-2, INF-γ, TNF-α, and TGF-β) were measured by ELISA at one month after treatment with MSCs. (**C**) After the MSC transfusion, the percentage of CD4^+^IFNγ^+^Th1 cells in samples of patient peripheral blood was analyzed by flow cytometry. (**D**) After the MSC transfusion, the percentage of CD4^+^IL-4^+^Th2 cells in samples of patient peripheral blood was analyzed by flow cytometry. (**E**) After the MSC infusion, the percentage of CD8^-^CD4^+^IL17A^+^Th17 cells in samples of patient peripheral blood was analyzed by flow cytometry. (**F**) After the MSC transfusion, the percentage of CD4^+^CD25^+^FOXP3^+^Treg cells in samples of patient peripheral blood was analyzed by flow cytometry. N = 15. Data represent the mean ± SEM. *P < 0.05, **P < 0.01, and ***P < 0.001.

Some demographic and clinical characteristics of the two study cohorts are summarized in Table 1. Fifteen chronic AA patients (8 males and 7 females; median age = 33 years) and 15 normal donors were recruited for this study. All patients received the same treatment for pre-transfusion conditioning. BMMSCs (mean number = 6 × 10^5^ mg/kg) were intravenously injected into each of the 15 chronic AA patients who had been refractory to prior immunosuppressive treatment. An analysis performed at one month after each MSC transfusion showed that the patient’s serum hemoglobin level had significantly increased. We also examined the percentages of Th1, Th2, Th17, and Treg cells and the levels of their associated cytokines (IL-2, INF-γ, TNF-α, and TGF-β) in serum and bone marrow at one month after each MSC transfusion, and found that the levels of IL-2 and IFN-γ (Th1/Th2 associated cytokines) were significantly reduced in both serum and bone marrow. Moreover, while the levels of TNF-α were reduced, the levels of TGF-β were significantly increased in both the serum and bone marrow of MSC-infused patients (Fig. 1A,B). An analysis of each patient’s peripheral blood revealed that the percentages of CD4^+^IFNγ^+^Th1 cells and CD8-CD4^+^IL-17A^+^Th17 cells were significantly decreased after the MSC transfusion (Fig. 1C–E), while the percentage of CD4^+^CD25^+^FOXP3^+^Treg cells was significantly increased (Fig. 1F).

### A Notch signaling-dependent pathway may modulate the differentiation of Th1, Th17, and Treg cells after a MSC transfusion in chronic AA patients

**Figure 2:**
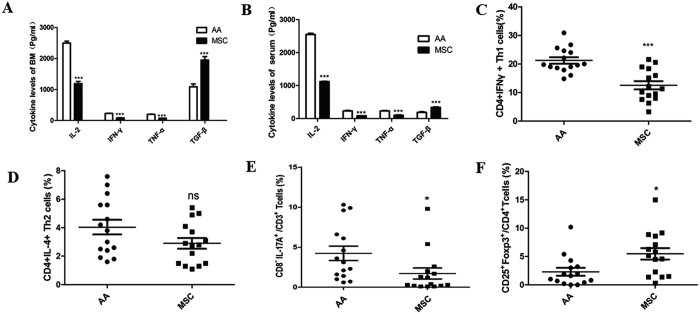
A Notch signaling-dependent pathway may have modulated Th1, Th17, and Treg cell differentiation in chronic AA patients who received a MSC transfusion. (**A**) Q-PCR analyses of Notch, Notch2, Notch3, and Notch4 expression in the PBMCs of patients treated with MSCs. (**B**) Q-PCR analyses of Dll1, Dll2, and Dll3 expression in the PBMCs of patients treated with MSCs. a (**C**) Q-PCR analyses of Jaggedl and Jagged2 expression in the PBMCs of patients treated with MSCs. (**D**) Q-PCR analyses of RBP-J, RORγT, and Foxp3 expression in the PBMCs of patients treated with MSCs. (**E**) Western blot analyses of Notchl, Notch2, Dll1, Jaggedl, RBP-J, RORγT, and Foxp3 expression in the PBMCs of patients. GAPDH was used as a loading control. (**F**) Densitometry plot of results shown in Fig. 2E. The relative expression levels were normalized to GAPDH. Data represent the mean ± standard error (n = 3). Data represent the mean ± SEM. *P < 0.05, **P < 0.01, ***P < 0.001.

Further in-depth study is required to gain a better understanding of how BMMSCs function in chronic AA patients. Previous studies have provided data concerning the differential roles played by Notch pathways in regulating Th1, Th2, Th17, and Treg cell differentiation^7^,^8^,^10^,^12^,^13^,^14^. We assessed the expression of Notch signaling receptors Notchl, Notch2, Notch3, and Notch4 in the PBMCs of AA patients after they received a MSC transfusion, and found that the levels of Notchl and Notch2 receptors were increased, while the levels of Notch3 and Notch4 receptors remained unchanged (Fig. 2A). Furthermore, the expression levels of Notch signaling ligands Dll1 and Jaggedl were enhanced, while no changes in Dll2, Dll3, and Jagged2 expression levels were found (Fig. 2B,C). We also assessed RBP-J, RORγt, and Foxp3 expression in the PBMCs of the patients, and found that the expression of all three transcription factors was significantly enhanced (Fig. 2D); however, a MSC infusion down-regulated RORγt expression. We next examined the levels of Notchl, Notch2, Dll1, Jaggedl, RBP-J, RORγt, and Foxp3 proteins in PBMCs of the patients, and found that a MSC infusion up-regulated Notchl, Notch2, Dll1, Jaggedl, RBP-J, and Foxp3 protein expression, but down-regulated RORγt protein expression (Fig. 2E,F). These results suggested that a Notch signaling-dependent pathway was involved in the modulation of Th1, Th17, and Treg cells by transfused MSCs in chronic AA patients.

### Notch/RBP-J/FOXP3/RORγt pathway was involved in modulating the Treg/Th17 ratio of the MSCs transfused *in vitro*

**Figure 3:**
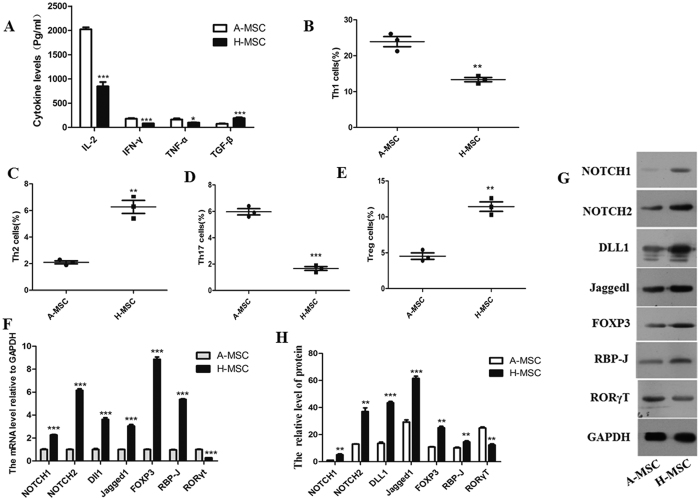
The Notch/RBP-J/FOXP3/RORγt pathway was involved in modulating the Treg/Th17 balance after MSC treatment *in vitro*. PBMCs were collected from patients, and the CD4+ lymphocyte subpopulation cells were isolated using antibody-coated immunomagnetic beads. Next, the isolated lymphocyte subpopulation cells were co-cultured with BMSCs from AA patients (A-MSCs) and donor-derived BMSCs (H-MSCs) at ratios of 10:1 for 4 days. (**A**) The concentrations of cytokines IL-2, INF-γ, TNF-α, and TGF-β in the culture supernatant were measured by ELISA. (**B**) After co-culturing the lymphocytes with BMSCs from AA patients (A-MSCs) and donor-derived BMMSCs (H-MSCs), the percentage of CD4 + IFNγ+ Th1 cells was analyzed by flow cytometry. (**C**) The percentage of CD4^+^ IL-4^+^Th2 cells was analyzed by flow cytometry (**D**) The percentage of CD8^−^CD4^+^IL-17A^+^Th17 cells was analyzed by flow cytometry. (**E**) The percentage of CD4^+^CD25^+^FOXP3^+^Treg cells was analyzed by flow cytometry. (**F**) Q-PCR analyses of Notchl, Notch2, Dll1, Jaggedl, RBP-J, RORγT, and Foxp3 expression in lymphocyte subpopulation cells after they were co-cultured with MSCs. (**G**) Western blot analyses of Notchl, Notch2, Dll1, Jaggedl, RBP-J, RORγT, and Foxp3 expression in lymphocyte subpopulation cells after they were co-cultured with MSCs. GAPDH was used as a loading control. (**H**) Densitometry plot of the results shown in Fig. 3G. The relative expression levels were normalized to GAPDH. Data represent the mean ± standard error (n = 3). *P < 0.05, **P < 0.01, ***P < 0.001.

PBMCs were collected from patients, and the CD4+ lymphocyte subpopulation cells were isolated using antibody-coated immunomagnetic beads. The isolated lymphocyte subpopulation cells were then co-cultured with BMMSCs from AA patients (A-MSCs) and donor-derived BMMSCs (H-MSCs) at ratios of 10:1 for 4 days; after which, the percentages of Th1, Th2, Th17, and Treg cells were detected by flow cytometry. The culture supernatant was collected, and its concentrations of cytokines IL-2, INF-γ, TNF-α, and TGF-β were measured. ELISA results showed that the levels of IL-2, INF-γ, and TNF-α in the H-MSC co-cultured supernatant were significantly decreased, while the TGF-β level was significantly increased (Fig. 3A). The percentages of Th1 and Th17 cells decreased significantly, while the percentages of Th2 and Treg cells increased after H-MSC co-culture (Fig. 3B–E). We next detected the levels of Notchl, Notch2, Dll1, Jaggedl, RBP-J, RORγt, and Foxp3 mRNA and protein expression in the lymphocyte subpopulation cells that had been co-cultured with A-MSCs and H-MSCs. H-MSC co-culture up-regulated the levels of Notchl, Notch2, Dll1, Jaggedl, RBP-J and Foxp3, but down-regulated the RORγt mRNA level (Fig. 3F). Similar results were seen when examining the corresponding protein levels (Fig. 3G,H). These results indicated that the infused MSCs modulated the levels of Th1, Th2, Th17, and Treg cells as well as their related cytokines by affecting the Notch/RBP-J/FOXP3/RORγt pathway.

### Infused MSCs regulated the Treg/Th17 balance in an AA mouse model by affecting the Notch/RBP-J/FOXP3/RORγt pathway

**Figure 4:**
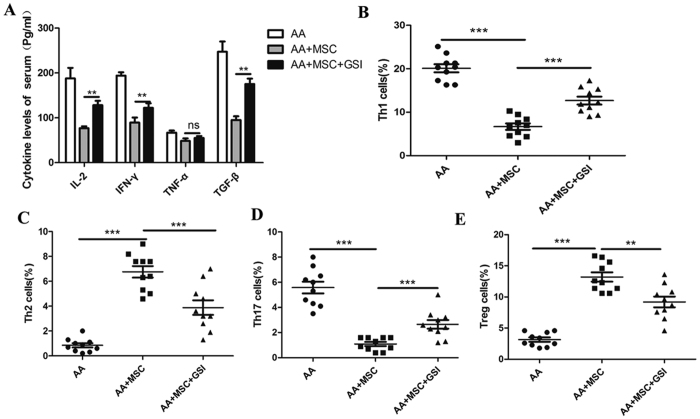
Treatment with MSCs regulated the Treg/Th17 balance via the Notch/ RBP-J/FOXP3/RORγt pathway in an AA mouse model. An AA mouse model was established, and the mice were randomly divided into three groups: AA group, AA + MSC group, and an AA + MSC + GSI group. AA + MSC group: mice were intravenously injected with MSCs (6 × 10^5^/kg) obtained from normal mice. AA + MSC + GSI group: mice were intravenously injected with MSCs (6 × 10^5^/kg) obtained from normal mice that had been injected with GSI. (**A**) The serum concentrations of IL-2, INF-γ, TNF-α, and TGF-β were measured by ELISA. (**B**) The percentage of Th1 cells among the mouse PBMCs was analyzed by flow cytometry. (**C**) The percentage of Th2 cells among the mouse PBMCs was analyzed by flow cytometry. (**D**) The percentage of Th17 cells among the mouse PBMCs was analyzed by flow cytometry. (**E**) The percentage of Treg cells among the mouse PBMCs was analyzed by flow cytometry, (n = 10). Data represent the mean ± SEM. *P < 0.05, **P < 0.01, ***P < 0.001.

After successfully establishing an AA mouse model (Table 2), the mice in the model were infused with BMSCs plus γ-secretase inhibitors (GSIs), which served to disrupt the Notch pathway. Decreased levels of IL-2, INF-γ, and TNF-α, and increased levels of TGF-β were found in the serum of the transfused mice. Moreover, these changes could be reversed by administration of GSI (Fig. 4A). A BMMSC transfusion decreased the percentages of Th1 and Th17 cells and increased the percentages of Th2 and Treg cells among the PBMCs of AA mice, when compared with the PBMC populations in control AA mice. GSI also significantly suppressed the ability of BMSCs to modulate the proportions of Th1, Th2, Th17, and Treg cells (Fig. 4B–E).

**Figure 5:**
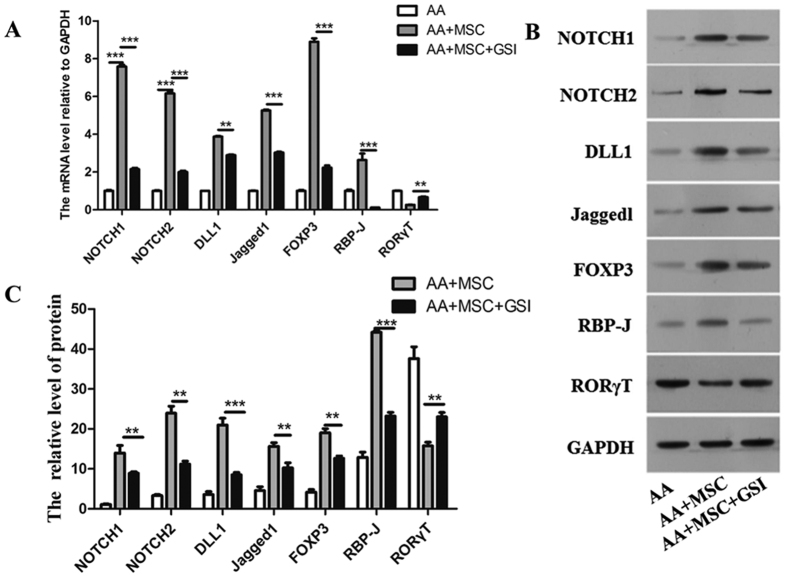
Treatment with MSCs regulated the Treg/Th17 balance via the Notch/ RBP-J/FOXP3/RORγt pathway in an AA mouse model. (**A**) Q-PCR analyses of Notchl, Notch2, Dll1, Jaggedl, RBP-J, RORγT, and Foxp3 expression in the PBMCs of mice (n = 10). (**B**) Western blot analyses of Notchl, Notch2, Dll1, Jaggedl, RBP-J, RORγT, and Foxp3 expression in the PBMCs of mice (n = 3). GAPDH was used as a loading control. (**C**) Densitometry plot of the results shown in Fig. 5B. The relative expression levels were normalized to GAPDH. Data represent the mean ± standard error (n = 3). Data represent the mean ± SEM. *P < 0.05, **P < 0.01, ***P < 0.001.

To further confirm how the Notch/RBP-J/FOXP3/RORγt pathway functions following an MSC transfusion, we examined the levels of mRNAs and proteins expressed by the pathway-related genes in the PBMCs of mice. As shown in Fig. 5A, a BMSC transfusion increased the levels of Notchl, Notch2, Dll1, Jaggedl, RBP-J, and Foxp3 mRNA and down-regulated the level of RORγT in the PBMCs, and these changes were reversed by GSI administration. Moreover, the Notch/RBP-J/FOXP3/RORγt pathway in mouse PBMCs was activated by a BMSC transfusion, and inhibited by GSI administration. The western blot results are shown in Fig. 5B,C. These findings suggest that in our AA mouse model, a MSC transfusion regulated the Treg/Th17 balance via a Notch signaling-dependent pathway.

## Discussion

The standard therapeutic options for chronic AA are immunosuppressive therapy with anti-thymocyte globulin (ATG) and cyclosporine (CsA) for older patients, or a bone marrow transfusion from a suitable donor^16^,^17^. Mesenchymal stromal cells (MSCs) isolated from bone marrow, adipose tissue, cord blood, and various fetal tissues are well known for their capacity to repair tissue, support hematopoiesis, and modulate immune and inflammation responses^18^,^19^. Previous studies showed that MSCs obtained from AA patients had a diminished capacity to form adherent cell layers, but did not display any accompanying changes in their typical morphology and mesenchymal markers. Furthermore, the proliferative and hematopoietic capacities of those MSCs were also diminished^20^. Moreover, BMMSCs from chronic AA patients displayed a reduced ability to stimulate T-cell activation and proliferation^21^,^22^. The collective data suggest that MSCs can reconstitute a damaged stomal layer and secrete an array of hematopoietic cytokines into a bone marrow microenvironment consisting of adipocytes, fibroblasts, osteoblasts, osteoclasts, and endothelial cells derived from MSCs. Previous studies showed that abnormal MSCs failed to regulate hematopoiesis, immune cell function, and niche^23^,^24^.

The feasibility and efficacy of performing a BMMSC transfusion to promote hematopoietic recovery have been demonstrated in a mouse model and several clinical studies. Human BM-MSCs secrete a variety of cytokines that support hematopoiesis in vivo and promote the engraftment of hematopoietic stem cells^25^,^26^,^27^,^28^. Allogeneic MSC transfusions have been safely performed in AA patients, and resulted in hematopoietic recovery^4^. However, Diego V *et al*.^28^ reported that a MSC transfusion had no effect in refractory or relapsed AA patients^29^. Overall, the effects of MSC transfusions in AA patients remain controversial and require further study.

In this study, the MSC transfusions significantly reduced the concentrations of cytokines IL-2, INF-γ, and TNF-α, and increased the TGF-β concentration in both serum and bone marrow. The percentages of Th1 and Th17 cells were significantly decreased after a MSC transfusion, while the percentage of Treg cells was significantly increased. Finally, a MSC transfusion alleviated the symptoms of anemia in refractory or relapsed AA patients and regulated their Th17/Treg cell balance.

Previous results showed increased numbers and frequencies of Th17 cells in both the BM and PBMCs of AA patients, and reduced numbers and frequencies of Treg cells. Th17 cells contribute to AA pathophysiology during the early stage of the disease^30^. Our results showed that the percentage of Th17 cells decreased in proportion to the concomitant increase in Treg cells. Further in-depth studies were conducted to gain a better understanding of the mechanism by which the Th17/Treg cell balance regulates BMMSCs in chronic AA patients. After analyzing the levels of Notch, Notch2, Notch3, Jaggedl, Jagged2, Notch4, RBP-J, RORγT, and Foxp3 expression, we found that the Notch/RBP-J/FOXP3/RORγt pathway was involved in regulating the Th17/Treg cell balance in chronic AA patients.

CD4+ lymphocyte subpopulation cells isolated from the PBMCs of patients were co-cultured with the BMMSCs from AA patients and donor-derived BMMSCs. Interestingly, the percentages of Th1 and Th17 cells, as well as the concentrations of cytokines IL-2, INF-γ, and TNF-α, were significantly decreased, while the percentages of Treg cells and concentrations of TGF-β were significantly increased. Additionally, we found that the Notch/RBP-J/FOXP3/RORγt pathway was involved in modulating the Treg/Th17 balance of the MSCs transfused *in vitro*. The role of transfused MSCs in regulating the Treg/Th17 balance via the Notch/RBP-J/FOXP3/RORγt pathway was further confirmed in an AA mouse model.

Bone marrow-derived MSCs are attractive for clinical use because they are relatively easy to collect and expand. Furthermore, they do not express major histocompatibility complex (MHC) class II or lymphocyte costimulatory molecules, and thus have low immunogenicity^31^. Our study confirmed the safety of performing an intravenous infusion of allogeneic MSCs into AA patients, and revealed the mechanism of action of those cells. Our results provide a valuable reference for designing additional clinical trials that use MSC transfusions as method of therapy. Such studies should be conducted prior to using MSC transfusions as therapy in large numbers of patients.

## Methods

### Mesenchymal stem cell preparation and transfusion

Fifteen chronic AA patients (8 males and 7 females; median age = 33 years) and 15 healthy donors were recruited for this study. All patients and healthy donors provided their written informed consent to participate in the study. Chronic AA was diagnosed based on criteria described at the Fourth National Aplastic Anemia Conference in 1987. All enrolled patients had either failed to respond to at least one previous first-line immunosuppressive therapy or relapsed afterwards. No patient had undergone hematopoietic stem cell transplantation prior to receiving the MSCs. A 5 mL sample of BM was aspirated from the posterior superior iliac crest of each donor subject; after which, the BMMSCs were isolated, cultured, and phenotypically characterized as per the standard protocol established in our laboratory^31^. Third passage cells were collected and immediately used as a fresh preparation. The transfusion recipients were treated with sodium bicarbonate, dexamethasone, and promethazine prior to receiving any human BMMSCs. A mean dose of 6 × 10^5^/kg MSCs was intravenously injected into each of the 15 chronic AA patients who had not responded to prior immunosuppressive treatment.

### Ethics statement

The experimental protocol was approved by the Ethics Committee of General Hospital of Guangzhou Military Command of Chinese PLA. All patients who participated in the study signed a written informed consent form. All experimental methods were carried out in accordance with the approved guidelines and regulations.

### Establishment of an AA Model and MSC Transfusion

BALB/c mice were obtained from the Guangdong Medical Lab Animal Center. All procedures performed on animals were carried out in accordance with the Guidelines for Human Treatment of Animals established by the Association of Laboratory Animal Sciences. Th**e** AA mouse model was established as previously described^30^,^32^,^33^. BALB/cBy mice were given a sublethal 5 Gy dose of total body radiation that was administered at a rate of ~1.0 Gy/min by a Model Cesium γ-irradiator (JL Shepherd & Associates; San Fernando, CA, USA) at one hour prior to a lymph node cell infusion. Inguinal, brachial, and axillary lymph node cells used for infusion were obtained from female DBA/2 mice, and infused into female BALB/cBy mice at a dose 1 × 10^6^ cells per recipient to induce AA.

### Isolation of PBMCs and lymphocyte subpopulation cells

Peripheral blood mononuclear cells (PBMCs) were isolated from AA patients, and CD4+ lymphocyte subpopulation cells were isolated by using antibody-coated immunomagnetic beads. Next, the CD4+ lymphocyte subpopulation cells were co-cultured with BMMSCs from AA patients (A-MSC) and donor-derived BMMSCs (H-MSCs) at ratios of 10:1 for 4 days; after which, the percentages of Th1, Th2, Th17, and Treg cells were determined by flow cytometry.

### Flow cytometry

Cells were separated, washed, resuspended, and stained with various antibody mixtures using methods described in the manufacturer’s instructions. When detecting Th1/Tc1 and Th17 cells, the cells were first incubated for 6 hours with phorbol myristate acetate (50 ng/mL) and ionomycin (750 ng/mL) in the presence of monensin at 37 °C. All human and mouse monoclonal antibodies were purchased from Becton Dickinson (Franklin Lakes, NJ, USA).

### ELISA

The amounts of IL-2, INF-γ, TNF-α, and TGF-β were detected with ELISA Kits (BioLegend, San Diego, CA, USA) according to the manufacturer’s instructions.

### RNA isolation and qRT-PCR

RNA isolation and qRT-PCR were performed as previously described^34^. Total RNA was isolated using Trizol reagent (Invitrogen; Carlsbad, CA, USA). A 1 μg sample of RNA was used as the template to synthesize single strand cDNA by using random primers and Primescript reverse transcriptase (M-MLV, Takara; Shiga, Japan) according to the manufacturer’s instructions. The primers used in this study are listed in Table 3. The cDNA was amplified by using SYBR green PCR Mix (iTAP, Bio-Rad) in conjunction with an Applied Biosystems (ABI) STEPONEPLUS sequence detection system (Applied Biosystems; Foster City, CA, USA). Following the analyses, fold-differences in results between groups were determined using the comparative cycle threshold (CT) method. Fold-changes were calculated from the ΔΔCT values, and using the formula 2−ΔΔCT.

### Western Blots

Cells were lysed in an ice-cold buffer (150 mM NaCl, 0.02% NaN_3_, 0.1% SDS, 50 mM TrisCl, pH 8.0, 100 μg/mL phenylmethylsulfonyl fluoride, 1 μg/mL aprotinin, and 1% Triton) for 30min; after which, the total protein concentration in the pooled lysates was measured using a BCA Protein Assay Kit (Pierce; Rockford, IL, USA). Next, samples of lysate containing 50 μg of protein were separated on a 12% SDS-PAGE gel, and the protein bands were transferred onto PVDF membranes (Millipore; Billerica, MA, USA). The PVDF membranes were then blocked with TBST buffer containing 2% BSA for 1 h; after which, they were incubated with antibodies specific for Notchl (1:1000, Santa Cruz Biotechnology; Santa Cruz, CA, USA), Notch2 (1:1000, Cell Signalling Technology; Danvers, MA), Dll1 (1:1000, Cell Signalling Technology), Jaggedl (1:1000, Santa Cruz Biotechnology), RBP-J (1:1000, Abcam; Cambridge, MA, USA), RORγT (1:1000, Abcam), and Foxp3 (1:1000, Cell Signalling Technology). The gels were then counterstained with HRP-conjugated goat anti-rabbit secondary antibodies (Promab Biotechnologies, 1:1000; Richmond, CA, USA), The individual protein bands were detected with the enhanced chemiluminescence (ECL) reaction (Kibbutz Beit Haemek, Israel), and the staining intensity of each band was quantified using Quantity One software (BioRad Laboratories; Hercules, CA, USA).

### Statistical Analysis

All results are presented as the mean ± S.E.M of data obtained from at least three independent experiments. The T-test was used to analyze parametric data and the Mann–Whitney U test was used to analyze non-parametric data. P-values < 0.05 were considered statistically significant.

## Additional Information

**How to cite this article:** Li, H. *et al*. In patients with chronic aplastic anemia, bone marrow–derived MSCs regulate the Treg/Th17 balance by influencing the Notch/RBP-J/FOXP3/RORγt pathway. *Sci. Rep.* **7**, 42488; doi: 10.1038/srep42488 (2017).

**Publisher's note:** Springer Nature remains neutral with regard to jurisdictional claims in published maps and institutional affiliations.

## Figures and Tables

**Table 1:** Baseline demographic and clinical characteristics of the study cohorts.

Variable	Patients	Donors
Number	15	15
Gender(female/male)	8/7	9/6
Age (years)	19–47	19–48
AA Classification	CAA	—
Body weight(kg)	61.3 ± 18.7	60.8 ± 20.1
Hemoglobin (g/dL)		
Before MSCT	7.1 ± 1.2	—
After MSCT	12.6 ± 1.7	—

MSCT, mesenchymal stromal cell transfusion. CAA, chronic AA.

**Table 2:** Blood counts in the AA mouse model.

	AA	Normal
Number	10	10
RBCs (10^12^/dL)	0.3 ± 0.1	1.4 ± 0.2
WBCs (10^8^/dL)	1.7 ± 1.2	11 ± 1.3
Hematocrit (%)	20 ± 6.1	57.3 ± 2.3
Platelets (10^10^/dL)	0.6 ± 0.2	5.8 ± 1.9
Reticulocytes (10^10^/dL)	1.2 ± 0.7	3.1 ± 1.2

RBCs, red blood cells; WBCs, white blood cells.

**Table 3:** Primers used in the study.

ID	Sequence (5′-3′)	bp
GAPDH F	CCTCGTCTCATAGACAAGATGGT	169
GAPDH R	GGGTAGAGTCATACTGGAACATG	
Hsa-Notch1 F	GAGGCGTGGCAGACTATGC	140
Hsa-Notch1 R	CTTGTACTCCGTCAGCGTGA	
Hsa-Notch2 F	CCTTCCACTGTGAGTGTCTGA	96
Hsa-Notch2 R	AGGTAGCATCATTCTGGCAGG	
Hsa-Dll1 F	GATTCTCCTGATGACCTCGCA	168
Hsa-Dll1 R	TCCGTAGTAGTGTTCGTCACA	
Hsa-Jagged1 F	GTCCATGCAGAACGTGAACG	136
Hsa-Jagged1 R	GCGGGACTGATACTCCTTGA	
Hsa-FOXP3 F	GTGGCCCGGATGTGAGAAG	238
Hsa-FOXP3 R	GGAGCCCTTGTCGGATGATG	
Hsa-RBP-J F	AACAAATGGAACGCGATGGTT	122
Hsa-RBP-J R	GGCTGTGCAATAGTTCTTTCCTT	
Hsa-RORγt F	CCTGGGCTCCTCGCCTGACC	169
Hsa-RORγt R	TCTCTCTGCCCTCAGCCTTGCC	
Hsa-Notch3 F	CGTGGCTTCTTTCTACTGTGC	122
Hsa-Notch4 F	GATGGGCTGGACACCTACAC	
Hsa-Notch4 R	CACACGCAGTGAAAGCTACCA	152
Hsa-Dll3 F	CGTCCGTAGATTGGAATCGCC	
Hsa- Dll3 R	TCCCGAGCGTAGATGGAAGG	82
Hsa-Jagged2 F	TGGGCGGCAACTCCTTCTA	
Hsa-Jagged2 R	GCCTCCACGATGAGGGTAAA	151

## References

[b1] Parmesar K. & Raj K. Haploidentical Stem Cell Transplantation in Adult Haematological Malignancies. Advances in hematology 2016, 3905907, doi: 10.1155/2016/3905907 (2016). 27313619 PMC4904087

[b2] Yamazaki H. [Acquired aplastic anemia]. [Rinsho ketsueki] The Japanese journal of clinical hematology 57, 91–97, doi: 10.11406/rinketsu.57.91 (2016). 26935624

[b3] Le Blanc K. & Ringden O. Mesenchymal stem cells: properties and role in clinical bone marrow transplantation. Current opinion in immunology 18, 586–591, doi: 10.1016/j.coi.2006.07.004 (2006). 16879957

[b4] Ball L. M. . Cotransplantation of ex vivo expanded mesenchymal stem cells accelerates lymphocyte recovery and may reduce the risk of graft failure in haploidentical hematopoietic stem-cell transplantation. Blood 110, 2764–2767, doi: 10.1182/blood-2007-04-087056 (2007). 17638847

[b5] Xiao Y. . Efficacy and safety of mesenchymal stromal cell treatment from related donors for patients with refractory aplastic anemia. Cytotherapy 15, 760–766, doi: 10.1016/j.jcyt.2013.03.007 (2013). 23731760

[b6] Li J. P., Zheng C. L. & Han Z. C. Abnormal immunity and stem/progenitor cells in acquired aplastic anemia. Critical reviews in oncology/hematology 75, 79–93, doi: 10.1016/j.critrevonc.2009.12.001 (2010). 20045349

[b7] Li M. O. & Rudensky A. Y. T cell receptor signalling in the control of regulatory T cell differentiation and function. Nature reviews. Immunology 16, 220–233, doi: 10.1038/nri.2016.26 (2016). PMC4968889 27026074

[b8] Omenetti S. & Pizarro T. T. The Treg/Th17 Axis: A Dynamic Balance Regulated by the Gut Microbiome. Frontiers in immunology 6, 639, doi: 10.3389/fimmu.2015.00639 (2015). 26734006 PMC4681807

[b9] Chen J., Yang J., Qiao Y. & Li X. Understanding the regulatory roles of natural killer T cells in rheumatoid arthritis: T helper cell differentiation-dependent or -independent? Scandinavian journal of immunology, doi: 10.1111/sji.12460 (2016). 27384545

[b10] Eisenstein E. M. & Williams C. B. The T(reg)/Th17 cell balance: a new paradigm for autoimmunity. Pediatric research 65, 26R–31R, doi: 10.1203/PDR.0b013e31819e76c7 (2009). 19218879

[b11] Fleming R. J. Structural conservation of Notch receptors and ligands. Seminars in cell & developmental biology 9, 599–607, doi: 10.1006/scdb.1998.0260 (1998). 9918871

[b12] Feng Y. H. & Tsao C. J. Emerging role of microRNA-21 in cancer. Biomedical reports 5, 395–402, doi: 10.3892/br.2016.747 (2016). 27699004 PMC5038362

[b13] Folini M. . miR-21: an oncomir on strike in prostate cancer. Molecular cancer 9, 12, doi: 10.1186/1476-4598-9-12 (2010). 20092645 PMC2823650

[b14] You P. . Jagged-1-HES-1 signaling inhibits the differentiation of TH17 cells via ROR gammat. Journal of biological regulators and homeostatic agents 27, 79–93 (2013). 23489689

[b15] Ziegler S. F. & Buckner J. H. FOXP3 and the regulation of Treg/Th17 differentiation. Microbes and infection 11, 594–598, doi: 10.1016/j.micinf.2009.04.002 (2009). 19371792 PMC2728495

[b16] Miano M. & Dufour C. The diagnosis and treatment of aplastic anemia: a review. International journal of hematology 101, 527–535, doi: 10.1007/s12185-015-1787-z (2015). 25837779

[b17] Zhao K. & Liu Q. The clinical application of mesenchymal stromal cells in hematopoietic stem cell transplantation. Journal of hematology & oncology 9, 46, doi: 10.1186/s13045-016-0276-z (2016). 27193054 PMC4870746

[b18] Kern S., Eichler H., Stoeve J., Kluter H. & Bieback K. Comparative analysis of mesenchymal stem cells from bone marrow, umbilical cord blood, or adipose tissue. Stem cells 24, 1294–1301, doi: 10.1634/stemcells.2005-0342 (2006). 16410387

[b19] Jin H. J. . Comparative analysis of human mesenchymal stem cells from bone marrow, adipose tissue, and umbilical cord blood as sources of cell therapy. International journal of molecular sciences 14, 17986–18001, doi: 10.3390/ijms140917986 (2013). 24005862 PMC3794764

[b20] Hamzic E., Whiting K., Gordon Smith E. & Pettengell R. Characterization of bone marrow mesenchymal stromal cells in aplastic anaemia. British journal of haematology 169, 804–813, doi: 10.1111/bjh.13364 (2015). 25819548

[b21] Bacigalupo A. . T-cell suppression mediated by mesenchymal stem cells is deficient in patients with severe aplastic anemia. Experimental hematology 33, 819–827, doi: 10.1016/j.exphem.2005.05.006 (2005). 15963858

[b22] Prasanna S. J., Gopalakrishnan D., Shankar S. R. & Vasandan A. B. Pro-inflammatory cytokines, IFNgamma and TNFalpha, influence immune properties of human bone marrow and Wharton jelly mesenchymal stem cells differentially. PloS one 5, e9016, doi: 10.1371/journal.pone.0009016 (2010). 20126406 PMC2814860

[b23] Le Blanc K. . Transplantation of mesenchymal stem cells to enhance engraftment of hematopoietic stem cells. Leukemia 21, 1733–1738, doi: 10.1038/sj.leu.2404777 (2007). 17541394

[b24] Fang B. . Cotransplantation of haploidentical mesenchymal stem cells to enhance engraftment of hematopoietic stem cells and to reduce the risk of graft failure in two children with severe aplastic anemia. Pediatric transplantation 13, 499–502, doi: 10.1111/j.1399-3046.2008.01002.x (2009). 18673358

[b25] Zhao Z. . Assessment of bone marrow mesenchymal stem cell biological characteristics and support hemotopoiesis function in patients with chronic myeloid leukemia. Leukemia research 30, 993–1003, doi: 10.1016/j.leukres.2005.12.010 (2006). 16448696

[b26] Wang H. . Co-transfusion of haplo-identical hematopoietic and mesenchymal stromal cells to treat a patient with severe aplastic. Cytotherapy 12, 563–565, doi: 10.3109/14653241003695059 (2010). 20380540

[b27] Lu L. L. . Isolation and characterization of human umbilical cord mesenchymal stem cells with hematopoiesis-supportive function and other potentials. Haematologica 91, 1017–1026 (2006). 16870554

[b28] Huang Y. L., Huang S. L. & Cai Y. [Effect of bone marrow mesenchymal stem cell infusion on hemato-poiesis in mice with aplastic anemia]. Zhongguo shi yan xue ye xue za zhi / Zhongguo bing li sheng li xue hui = Journal of experimental hematology / Chinese Association of Pathophysiology 15, 1005–1008 (2007). 17956679

[b29] Cle D. V. . Intravenous infusion of allogeneic mesenchymal stromal cells in refractory or relapsed aplastic anemia. Cytotherapy 17, 1696–1705, doi: 10.1016/j.jcyt.2015.09.006 (2015). 26589752

[b30] de Latour R. P. . Th17 immune responses contribute to the pathophysiology of aplastic anemia. Blood 116, 4175–4184, doi: 10.1182/blood-2010-01-266098 (2010). 20733158 PMC2993623

[b31] Singh S. P., Tripathy N. K. & Nityanand S. Comparison of phenotypic markers and neural differentiation potential of multipotent adult progenitor cells and mesenchymal stem cells. World journal of stem cells 5, 53–60, doi: 10.4252/wjsc.v5.i2.53 (2013). 23671719 PMC3648646

[b32] Chen J. . Minor antigen h60-mediated aplastic anemia is ameliorated by immunosuppression and the infusion of regulatory T cells. Journal of immunology 178, 4159–4168 (2007). 10.4049/jimmunol.178.7.4159 17371972

[b33] Bloom M. L. . A mouse model of lymphocyte infusion-induced bone marrow failure. Experimental hematology 32, 1163–1172, doi: 10.1016/j.exphem.2004.08.006 (2004). 15588941

[b34] Ren J. . Estrogen upregulates MICA/B expression in human non-small cell lung cancer through the regulation of ADAM17. Cellular & molecular immunology 12, 768–776, doi: 10.1038/cmi.2014.101 (2015). 25363527 PMC4716616

